# Preventive Prosthodontics: Integrating Preventive Prosthodontic Principles Into the Indian Oral Health Policy Framework

**DOI:** 10.1155/ijod/7265763

**Published:** 2026-10-20

**Authors:** Bondada Venkata Mani Anirudh, Rodrigues Shobha Janette, Mundathaje Mahesh

**Affiliations:** ^1^ Department of Prosthodontics Manipal College of Dental Sciences Mangalore Manipal Academy of Higher Education Manipal Karnataka India manipal.edu

**Keywords:** disease prevention, edentulism, healthy aging, oral health policy, preventive prosthodontics, prosthodontic maintenance, public oral health

## Abstract

**Background:**

Prosthodontics is considered to be a rehabilitative discipline that aims at improving the functional, esthetic, and quality aspects of an individual’s mouth after the loss of their teeth. Recent research indicates that prosthodontics can also play a preventive role by preserving oral structures, timely intervention, maintenance and prevention of biological and mechanical problems related to prosthodontic rehabilitation. Despite its clinical relevance, the concept of preventive prosthodontics remains underrepresented within Indian oral health policy frameworks.

**Objective:**

The purpose of this narrative review is to discuss in depth the concept of preventive prosthodontics, analyze the importance of preventive prosthodontics in the preservation of oral structures, maintaining oral function, prevention of biological and mechanical complications associated with prosthetic rehabilitation, and supporting long‐term patient outcomes across the primary, secondary, and tertiary levels of prevention. The review further evaluates the integration of these preventive principles into the Indian oral health policy framework.

**Methods:**

A narrative review was carried out following the criteria recommended by Scale for Assessment of Narrative Review Articles (SANRA). Electronic databases such as PubMed, Scopus, Google Scholar, and Cochrane library were searched for relevant literature in the years ranging from January 2000 to January 2026 through combinations of the keywords preventive prosthodontics, prosthodontic maintenance, prosthodontic complications, oral health policy, levels of prevention, and minimally invasive dentistry. Relevant documents from the policies initiated by WHO and Indian government for oral health were also considered. After considering their relevance to the theme, 33 articles and documents were selected.

**Results:**

It was observed that the key elements of preventative prosthodontics include the use of preservation‐based treatment plans, structured recall and maintenance protocols, educating patients regarding hygiene practices to take care of the prosthesis, and dealing with biological and mechanical problems. These techniques were invariably in agreement with the concept of primary, secondary, and tertiary prevention and always showed improvement in the longevity of the prosthesis, retention of oral functions, and lessening of treatment burden. It was also noted that there may be significant scope for integrating these techniques into existing Indian oral health initiatives such as the National Oral Health Program (NOHP) and Ayushman Bharat Health and Wellness Centers (HWCs).

**Conclusions:**

Preventive prosthodontics can be considered an overlooked aspect that has the potential to connect clinical prosthodontics and oral health in general population. Understanding the importance of its preventive approach can contribute toward promoting healthy aging and functional benefits, as well as aid in incorporating preventive aspects of prosthodontics in education, clinical practice, and oral health policy of India. Further clinical and policy‐oriented research is needed to strengthen the evidence base and guide implementation across different healthcare settings.

## 1. Introduction

Oral diseases are still some of the most common noncommunicable diseases around the world and contribute significantly to the public health problem due to their high prevalence, chronicity, and effects on the general well‐being of an individual. Dental caries, periodontal disease, and tooth loss have continued to affect millions of people all around the globe, leading to problems with mastication, speech, appearance, psychosocial effects, and reduced quality of life [1–4]. Edentulism, which is viewed as the ultimate result of progressive development of dental diseases, leads to functional impairment, poor nutrition, and a decline in overall well‐being, especially in elderly and socioeconomically disadvantaged populations [3, 4]. In the context of increasing life expectancy and population aging, preservation of functional dentition and maintenance of oral function throughout the life course have emerged as major global oral health priorities [5, 6].

Prosthodontics has traditionally played a central role in restoring oral function and esthetics through the rehabilitation of missing teeth and associated oral structures using fixed, removable, and implant‐supported prostheses. Advances in biomaterials, digital workflows, and implant prosthodontics have substantially improved treatment predictability and patient‐centered outcomes. Nevertheless, prosthodontic rehabilitation continues to face important biological and mechanical challenges, including secondary caries, periodontal breakdown, prosthesis fracture, loss of retention, peri‐implant mucositis, and peri‐implantitis, all of which may compromise long‐term treatment success [7–9]. Prosthodontic rehabilitation has also been shown to improve masticatory function and oral health‐related quality of life in edentulous patients, with some studies showing that nutritional status can be improved with prosthetic treatment and dietary counseling [10]. Increasing evidence suggests that successful prosthodontic care depends not only on the placement of a prosthesis but also on sustained maintenance, early detection of complications, and preservation of supporting oral tissues over time.

Even with the development of new prosthetic materials, digital technology, and implant dentistry, most of the prosthodontic care still follows the rehabilitative paradigm, where therapy starts once teeth have already been damaged or lost. While the approach works for restoring the function of the mouth, there is little concern for preventing the development of problems, preserving the remaining oral tissues, and sustaining the results of the procedure. With the change in the perspective of patients, who now expect to age healthily, prosthodontic care is starting to be understood not as a single restorative intervention but as a continuous process that includes prevention, maintenance, and regular follow‐up.

Restorative philosophies of today have moved toward interception as well as minimally invasive methods, which stress on preserving existing tooth structures and intervene early so that comprehensive restoration does not become essential. The interceptive treatment philosophy by Dietschi incorporates the element of prevention at all stages of the treatment sequence starting from risk factor control and interceptive treatment to restoration treatment, regeneration, and replacement, if and when required. This preservation‐oriented philosophy is consistent with the preventive prosthodontics, which too stresses on preserving the oral tissues and preventing their diseases [11].

From these ideas for preservation, preventive prosthodontics extends the idea of traditional prosthodontics by broadening the concept from being purely replacement‐oriented to include the idea of tissue preservation, reduction of the risk of future diseases, and long‐term oral function maintenance. Preventive prosthodontics utilizes ideas including preservation of strategically valuable teeth, occlusal balance, designs for prostheses that will allow effective hygiene, education of patients, recall programs, and treatment of biological and mechanical complications. These ideas can be seen in relation to the concept of primary, secondary, and tertiary prevention used in public health to frame prosthodontics not just as a rehabilitative field but preventive field as well [12].

Contemporary oral health policy increasingly advocates prevention‐oriented, integrated, and life‐course approaches to oral healthcare. Global initiatives such as the World Health Organization Global Oral Health Strategy (2023–2030), together with national programs aimed at strengthening primary healthcare and universal health coverage, emphasize prevention, continuity of care, and maintenance of functional health outcomes across the lifespan [13, 14].

Even though the World Health Organization’s Global Strategy and Action Plan for Oral Health 2023–2030 does not specifically recognize preventive prosthodontics as an important part of the policy on oral health, it stresses the importance of prevention, integration of oral health services into primary health care, person‐centered care, healthy aging, and rehabilitation. These areas correlate well with the basic concepts of preventive prosthodontics, including maintaining functionality, providing continuity of care, and avoiding complications in the future. Preventive prosthodontics may thus be regarded as an approach that supports the broader objectives of the WHO strategy, even though it is not specifically identified within the policy document.

In India, oral health policies such as the National Oral Health Program (NOHP), Ayushman Bharat Health and Wellness Centers (HWCs), and Ayushman Bharat–Pradhan Mantri Jan Arogya Yojana (PM‐JAY) have strengthened the emphasis on prevention, early detection, and improving access to oral healthcare. However, these initiatives mainly focus on disease prevention and treatment services, with little explicit attention given to prosthodontic maintenance, structured recall protocols, or the long‐term preservation of oral function following prosthodontic rehabilitation. Incorporating these preventive aspects into existing policy frameworks could strengthen continuity of care and improve long‐term prosthodontic outcomes [15, 16].

Despite these changes; however, the concept of prosthodontics is still largely perceived in the healthcare context as a restorative or replacement technique, with much less attention given to prosthodontic maintenance, preventing complications associated with prosthodontics, and maintaining oral function as a critical part of preventive care.

This disconnection between the current oral healthcare policy and the preventive possibilities offered by prosthodontics is indeed one that must be addressed. Although the principles of prevention have been incorporated into restorative dentistry, periodontology, and public oral health, their systematic implementation in the field of prosthodontics is far from being achieved. Understanding prosthodontics as a preventive discipline will help alleviate the burden of treatment, prolong prosthesis life, protect oral structures, and promote healthy aging through oral function.

Accordingly, this narrative review aims to provide a comprehensive overview of preventive prosthodontics by discussing its role in preserving oral structures, maintaining oral function, preventing prosthesis‐related biological and mechanical complications, and supporting long‐term patient outcomes across the primary, secondary, and tertiary levels of prevention. The review further explores the integration of these preventive principles into the Indian oral health policy framework within the broader context of contemporary oral health policy.

## 2. Materials and Methods

### 2.1. Review Design

The current review was carried out as a narrative review of the current evidence on preventive prosthodontics and its relevance to oral health policy and disease prevention. The current review was designed according to the recommendations of the Scale for Assessment of Narrative Review Articles (SANRA) in order to increase the methodology of the narrative evidence synthesis. An evaluation of the current review using the SANRA scale is presented in Table S1.

### 2.2. Search Strategy

A thorough electronic search of the literature was conducted utilizing the following databases: PubMed, Scopus, Google Scholar, and Cochrane Library. The search was based on literature published from January 2000 to January 2026 to account for the evolution of preventive dentistry, prosthodontic rehabilitation, maintenance approaches, and policies for oral health care management.

Medical subject headings (MeSHs) terms and free keywords were employed in the search process. Key terms used in the literature search include preventive prosthodontics, prosthodontic maintenance, prosthodontic complications, oral health policy, prevention levels, prosthesis longevity, minimally invasive dentistry, and prevention of edentulism. Boolean operators (AND and OR) were used in the search.

### 2.3. Additional Sources

In order to enhance the policy‐related aspects of the review, certain gray literature and policy documents were considered as supporting sources of information for the discussion. This included the World Health Organization publications, Ministry of Health and Family Welfare (India), NOHP, Ayushman Bharat/PM‐JAY, and National Program for Health Care of the Elderly (NPHCE). The references of the eligible documents were also searched manually for other sources. These sources provided the context of the concept and were not included in the narrative synthesis if they did not meet the eligibility criteria.

### 2.4. Eligibility Criteria

Articles were considered eligible if they addressed one or more of the following domains: preventive approaches within prosthodontic care; maintenance protocols for fixed, removable, or implant‐supported prostheses; prevention and management of biological and mechanical complications; preservation‐oriented treatment planning; or integration of prosthodontic care within public health and policy frameworks.

The included sources comprised original research articles, systematic reviews, narrative reviews, consensus reports, policy papers, and relevant governmental and international health reports published in English. Studies focused exclusively on laboratory or material science aspects without direct preventive clinical implications were excluded. Isolated case reports lacking broader preventive relevance, conference abstracts without full‐text availability, and duplicate publications were also excluded from the review.

The search was limited to English‐language publications to ensure consistency in data interpretation and because translation resources were not available. This approach may have resulted in the exclusion of relevant evidence published in other languages. The inclusion and exclusion criteria used for study selection are summarized in Table 1.

**Table 1:** Eligibility criteria for study selection.

Criterion	Inclusion criteria	Exclusion criteria
Study design	Narrative reviews, systematic reviews, clinical studies, observational studies, consensus statements, policy documents, and relevant expert reports	Editorials, letters to the editor, conference abstracts without full text, opinion pieces lacking supporting evidence
Topic	Studies addressing preventive prosthodontics, prosthodontic maintenance, prosthesis‐related complications, recall protocols, oral health policy, prevention, rehabilitation, and continuity of care	Studies unrelated to prosthodontic prevention or long‐term maintenance (e.g., purely laboratory material testing without clinical or preventive relevance)
Population	Adults and older adults receiving prosthodontic care, including complete dentures, removable partial dentures, fixed prostheses, and implant‐supported prostheses	Animal studies and in vitro studies not relevant to the review objectives
Language	English	Languages other than English
Publication period	January 2000–January 2026	Publications outside the selected time frame

### 2.5. Study Selection and Synthesis

The primary literature search resulted in roughly 160 entries in the chosen databases. Following the identification and elimination of irrelevant titles and abstracts, 65 sources were selected for further examination of their full texts. Of the selected 65 papers, 33 were finally included in the narrative synthesis based on their thematic significance and eligibility. The summary of the literature search process is shown in the flow diagram (Figure 1) in accordance with the PRISMA statement. Apart from the selected literature, policy documents, international guidelines, and background references used for contextual support were also cited. However, these references did not participate in the literature synthesis itself.

**Figure 1:**
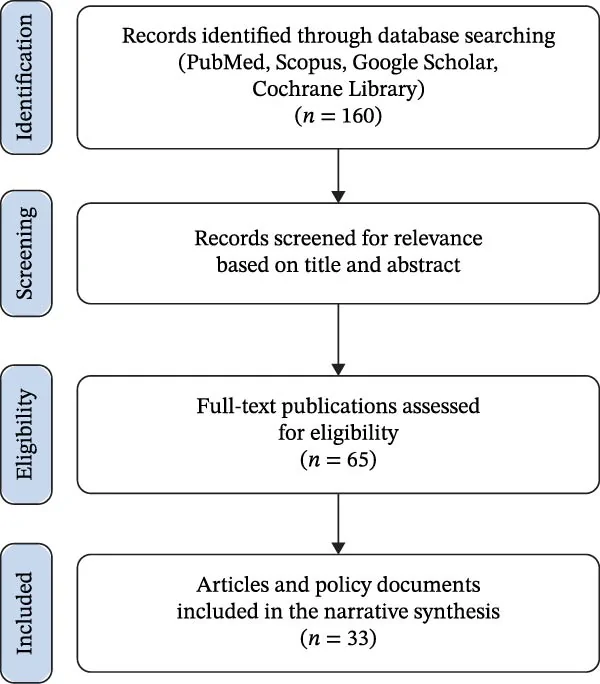
PRISMA‐style flow diagram illustrating the identification, screening, eligibility assessment, and final inclusion of studies in the present narrative review.

Study selection was performed by a single reviewer using predefined eligibility criteria. As this was a narrative review, independent duplicate screening and inter‐reviewer agreement statistics were not applied.

A thematic narrative synthesis approach was adopted. The included literature was analyzed and organized under the following conceptual domains:

1. Concept of preventive prosthodontics
2. Preventive prosthodontics across levels of prevention
3. Integration into oral health policy and healthcare systems
4. Challenges and barriers to implementation
5. Future directions for policy and clinical practice

### 2.6. Rationale for Narrative Review Approach

Given the conceptual, interdisciplinary, and policy‐oriented nature of preventive prosthodontics, a narrative review methodology was considered appropriate to integrate evidence from clinical prosthodontics, preventive dentistry, public health policy, and health systems literature into a comprehensive conceptual framework.

## 3. Concept of Preventive Prosthodontics

The concepts discussed in this section are derived from the recurring themes identified across the 33 publications included in the narrative synthesis and are presented as an evidence‐informed conceptual overview of preventive prosthodontics.

The included literature conceptualizes preventive prosthodontics as an evolving concept that incorporates the application of preventive concepts through all stages of prosthodontic rehabilitation [12, 17]. Rather than focusing exclusively on the replacement of missing teeth, this approach concentrates on maintaining the health of residual dental structures, identifying risk factors, adopting minimally invasive treatment where appropriate, and long‐term maintenance in order to maintain oral health and function [11, 18]. Therefore, preventive prosthodontics not only includes the provision of prostheses but also includes tooth conservation, periodontal and peri‐prosthetic health, education of the patients, follow‐up, and prevention of any biological and mechanical problems during the lifespan of the prosthesis [12, 17].

Preventive prosthodontics has emerged due to the growing recognition that the success of prosthodontic rehabilitation is not solely based on the success of prosthesis survival. Long‐term clinical studies and systematic reviews have shown that even in the presence of good survival rates of both tooth‐ and implant‐retained prostheses, biological complications such as caries, periodontal and peri‐implant disease, along with mechanical problems including loss of retention, veneer fracture, screw loosening, and framework failures can occur. These findings indicate that prosthodontics is not just about providing a prosthesis but requires maintenance and periodic evaluation of the prosthesis and timely management of complications to preserve oral health, increase prosthesis longevity, and functional outcomes [7, 19–21].

The included literature consistently describes preventive prosthodontics as a model of care that is preventive in nature, patient‐focused, and involves continuous monitoring [12, 17, 18]. In contrast to the traditional approach to seeing rehabilitation as one form of restoration, this particular philosophy stresses the need for preserving the vital teeth and oral structures, maintaining periodontal and peri‐implant health, optimizing the relationship between the occlusion, design of the prosthesis for good plaque control, risk assessment, recall programs, patient education, and managing biological and mechanical complications before they lead to total prosthesis failure [21–24]. Current evidence indicates that maintenance planning should begin during treatment planning rather than after prosthesis delivery, with follow‐up periods based on the individual risk profile of the patient. Moreover, the prosthetic design itself can serve as a preventive approach because of cleansable emergence profiles, the hygienic pontics, the appropriate contouring of the removable partial denture framework, and the use of restorations that make it possible to provide adequate hygiene, all contribute to the prevention of disease and help maintain periodontal/peri‐implant tissues in good condition [21, 25, 26]. In total, these philosophies correlate with the concept of minimally invasive and evidence‐based dentistry, which stresses the importance of preservation of healthy tissue, individualized preventive care, and maintenance of oral function instead of complicated restorative replacement [11, 18, 27].

Beyond improving longevity of prosthesis, preventive prosthodontics is beneficial to other goals that concern patients’ and public health care [3, 28]. By preserving functional dentition, maintaining the periodontal and peri‐implant health, and promoting long‐term longevity of prostheses will ensure efficient mastication, proper nutrition, improved speech and phonetics, preservation of occlusal harmony and stomatognathic functions, facial esthetics, psychosocial welfare, and overall oral health‐related quality of life, especially among older adults and medically compromised patients [3, 10]. These outcomes can be considered close to the life‐course approach to oral health suggested by the World Health Organization, where maintenance of function and healthy aging is preferable to disease management. Moreover, by decreasing the biological and mechanical issues associated with prostheses, delaying the need for complicated retreatment procedures, and preserving existing oral tissues, preventive prosthodontic techniques can lower overall healthcare costs and enhance sustainability [28, 29]. Overall, these additional advantages emphasize the significance of preventive prosthodontics as an important part of modern oral care.

In summary, the collected evidence indicates that preventive prosthodontics can be considered a comprehensive philosophy of care as opposed to merely being an individual clinical procedure or routine maintenance. Incorporation of preventive aspects into the treatment plan, design of the prosthesis, rehabilitation process, and follow‐up expands the traditional rehabilitation of prosthodontics from simply reconstructive to preserving the integrity of oral tissues, maintaining their function, and preventing disease in the future. Thus, preventive prosthodontics becomes a concept linking restorative dentistry to contemporary public health priorities.

## 4. Levels of Prevention and Preventive Interventions in Prosthodontics

The principles of preventive prosthodontics can be effectively understood within the classical public health framework of primary, secondary, and tertiary prevention. Application of this preventive hierarchy to prosthodontic care broadens the traditional restorative scope of the specialty by emphasizing the preservation of oral tissues, early interception of disease processes, maintenance of prosthetic function, and prevention of long‐term biological and mechanical complications. This framework further positions prosthodontics as a longitudinal continuum of care rather than an isolated rehabilitative intervention. The preventive strategies discussed under each level are derived from the recurring themes identified in the included literature and are supported by the representative studies cited within each subsection.

### 4.1. Primary Prevention

According to the included literature, the main focus of primary prevention in prosthodontics is the preservation of natural dentition and oral structures. At this stage, preventive measures are aimed at preventing tooth loss and any need for future prosthetic treatments through the control of modifiable risk factors prior to permanent changes. It is important to note that this approach does not only focus on disease prevention but also on tooth preservation [12, 17, 30].

The preventive measures can be listed as the following: reinforcement of oral hygiene instructions, nutrition counseling, caries prevention, periodontal disease control, management of parafunctional habits at an early stage, occlusal equilibration, and retention of strategically valuable teeth in order to maintain the arch integrity and occlusion [12, 17, 24]. The contemporary treatment planning in prosthodontics emphasizes more on conservative and noninvasive methods, such as adhesive restoration concepts, overdenture abutment preservation, and retention of salvageable teeth. Prosthodontic interventions described in the included literature are preventive implant treatment planning, stents in the case of head and neck radiotherapy, and protection of oral cavity with the help of the mouthguards. These measures will contribute to the maintenance of oral structures and decrease the complexity of future rehabilitation [21, 25, 26]. Such a strategy will prevent unnecessary tooth extraction and help maintain the alveolar bone, proprioception, and oral functions. Thus, the burden of prosthetic rehabilitation can be significantly decreased.

### 4.2. Secondary Prevention

Across the included literature, secondary prevention in prosthodontics is always characterized by systematic maintenance and early management of biological and mechanical complications. This level of prevention is not concerned with simply placing a prosthesis; rather, it involves ongoing monitoring and follow‐up for complications that could lead to irreparable harm to tissues or a failed prosthesis [21, 26].

The preventive strategies at this stage involve clinical inspection of prostheses and supporting structures, radiographic evaluation if required, analysis of occlusion, prosthesis retention and stabilization, and reinforcing oral hygiene practices. Prompt detection of marginal discrepancies, prosthesis wear, fracture lines, soft tissue irritation, inflammation around abutments, peri‐implant mucositis, loss of retention, and occlusal instability allows conservative management by means of occlusal adjustment, occlusal splints for bruxism, relining, rebasing, prosthesis repair, hygiene improvement, and management of local inflammation [19, 20, 25, 30, 31]. Conservative treatment can often avoid the need for extensive prosthesis replacement, maintain the health of the supporting structures of the mouth, and minimize the costs of subsequent treatment.

In the reviewed literature, systematic maintenance protocols and the prompt treatment of biological and mechanical problems always corresponded to increased prosthetic longevity, maintenance of oral tissue support, and decreased need for retreatments. This supports the idea that secondary prevention is the essential bridge connecting prosthodontic treatment and functional stability [7, 20, 21, 26].

### 4.3. Tertiary Prevention

The included literature identifies the preservation of oral function and limitation of disability as the principal objectives of tertiary prevention in prosthodontics. In this context, preventative measures are directed toward patients who have prosthodontic complications or need complex rehabilitation procedures. Tertiary prevention involves stabilization of the existing situation and prevention of further complications rather than merely replacing the failed prosthesis [3, 21].

Some examples of tertiary preventive procedures as found in literature are the use of an interim prosthesis that help to maintain the continuity of the arch while doing staged rehabilitation; overdenture treatment in order to maintain the residual alveolar bone and proprioception; preservation‐oriented prosthodontic treatment planning; preventive implant treatment with an objective to decrease ridge resorption; partial extraction treatments like root submergence and socket shield procedure; and finally maxillofacial prosthetic rehabilitation to preserve the functions after surgical procedures of ablation. The main goal of all these measures is the preservation of existing oral structures and their functionality and avoiding repetitive complex treatments [20, 30–32].

Tertiary preventive care goes beyond replacing failed prosthesis and focuses on maintaining functionality, adequate nutrition, speech ability, psychosocial well‐being, and oral health‐related quality of life. The patient‐focused approach to tertiary prevention is especially relevant for elderly, compromised medical patients, and those who need complicated prosthodontic or maxillofacial rehabilitation since the preservation of functionality is more attainable and useful than the replacement of prostheses [26, 29]. The preventive prosthodontic interventions across the primary, secondary, and tertiary levels of prevention and their corresponding policy linkages are summarized in Table 2.

**Table 2:** Preventive prosthodontic interventions across the levels of prevention and their alignment with oral health policy.

Level of prevention	Preventive objective	Prosthodontic interventions	Expected outcomes	Health system/policy linkage
Primary prevention	Prevent or delay prosthodontic need	Oral hygiene reinforcement; dietary counseling; caries and periodontal control; preservation of strategically important teeth and overdenture abutments; minimally invasive restorative approaches; preventive implant treatment planning; radiotherapy stents and protective oral appliances where indicated.	Reduced tooth loss; preserved alveolar bone; maintenance of natural dentition; delayed prosthodontic dependency	National Oral Health Program (NOHP); community oral health programs; preventive dental services; primary oral healthcare initiatives
Secondary prevention	Early detection and management of developing prosthodontic complications	Structured recall visits; prosthesis surveillance; radiographic monitoring where indicated; occlusal assessment; occlusal splint therapy for parafunctional habits where indicated; relining/rebasing; repair of minor prosthetic defects; peri‐implant and periodontal monitoring; reinforcement of hygiene practices	Improved prosthesis longevity; reduced biological and mechanical complications; early complication control; lower retreatment burden	Ayushman Bharat Health and Wellness Centers (HWCs); recall‐based care pathways; primary healthcare screening models; continuity‐of‐care systems
Tertiary prevention	Limit disability and preserve long‐term oral function	Management of established prosthodontic complications; repair, relining, or redesign of prostheses; management of peri‐implant disease; preservation‐oriented prosthodontic treatment planning; individualized maintenance protocols; nutritional and functional rehabilitation.	Sustained oral function; preservation of residual tissues; improved quality of life; reduced repeated complex rehabilitation	National Program for Health Care of the Elderly (NPHCE); specialist referral networks; rehabilitation‐centered public health services
Policy integration	Embed preventive prosthodontics within oral health systems	Prosthodontic maintenance protocols; reimbursement for recall care; integration into geriatric and primary care services; inclusion in oral health indicators and surveillance	Sustainable oral healthcare delivery; improved equity; cost reduction; healthy aging support	NOHP; Ayushman Bharat; PM‐JAY; Universal Health Coverage frameworks; WHO life‐course oral health strategy

### 4.4. Prosthodontics Across the Prevention Continuum

The included literature demonstrates that prosthodontics involves much more than just tooth replacement. Preventive prosthodontics is a process that goes throughout the entire disease spectrum—from the conservation of natural teeth and risk factor modification to the prevention of complications related to prosthodontics and post‐rehabilitation maintenance. Such a broader view on prevention will help develop an appropriate framework of prosthodontic integration into overall dental health care [28, 33].

## 5. Integration of Preventive Prosthodontics Into Oral Health Policy and Health Systems

There are wider considerations for preventive prosthodontics that go far beyond chairside clinical application into the larger realms of dental health policy, healthcare provision, and health care management of populations. Although prosthodontics has traditionally been positioned within healthcare systems as a rehabilitative specialty focused on replacement of lost oral structures, its preventive aspects coincide very much with the current public health agenda of disease prevention, continuity of care, healthy aging, and preservation of functional health outcomes throughout life [13, 14]. Recognition of prosthodontics within this preventive framework creates opportunities to integrate long‐term prosthodontic maintenance, structured follow‐up, and complication prevention into broader oral healthcare systems.

Among the many possibilities that exist regarding integrating prosthetics into oral health care initiatives, one clear example may be found in the NOHP of India, which places an emphasis on preventive and promotive oral healthcare. Although current programs for improving oral health primarily involve preventive approaches such as reducing disease burden through education, screening, and basic preventive services, relatively little attention is paid to maintaining prosthetic rehabilitation and maintaining long‐term functionality after treatment. The introduction of preventive approaches in prosthodontics, involving the use of a recall system, patient education on the maintenance of prostheses, as well as regular evaluation of the prosthesis’ functionality, and early management of prosthesis‐related complications, could expand the preventive scope of the program beyond disease prevention alone toward sustained oral rehabilitation and functional preservation [15]. Such an approach may be particularly relevant in populations with an increasing burden of partial and complete edentulism.

Ayushman Bharat HWCs, at the level of primary healthcare facilities, serve as a crucial vehicle for implementing preventive prosthodontics by way of community‐based screening, referrals, and continuity of care. These facilities are already established as the initial point of access for delivering preventive and promotive healthcare. The incorporation of basic prosthodontic monitoring activities like assessing the function of dentures, assessing the condition of the oral soft tissues, screening for mucosal conditions caused by the prosthesis, and reinforcing patient education on maintenance can help identify potential issues and refer patients to secondary or tertiary prosthodontic treatment when required. Such an approach will ensure that prosthodontic rehabilitation is a continuous preventive activity integrated into primary healthcare delivery. Health‐financing models also provide a critical avenue for advancing preventive prosthodontics. Under Ayushman Bharat–PM‐JAY, policy discussions surrounding oral healthcare have largely focused on treatment access and financial protection for major procedures, while maintenance‐oriented outpatient preventive services remain comparatively underemphasized [16].

While specific reimbursement for preventive maintenance of prosthodontics is rarely provided, there has been the inclusion of aspects of preventive care in the present healthcare financing schemes. In the case of Germany, for instance, the statutory health insurance connects regular preventive visits to the dentist with a greater reimbursement of dental prostheses by a system of bonuses. Those patients who visit their dentists regularly will get more fixed reimbursement for their prosthodontics treatments [34]. This kind of incentive model of reimbursement can show how preventive actions can be tied to prosthodontic reimbursement.

Nevertheless, structured follow‐up care, routine prosthodontic maintenance visits, early management of biological and mechanical complications, and preventive monitoring of implant‐ and tooth‐supported prostheses may reduce the need for repeated complex rehabilitation and lower long‐term treatment expenditure. From a health economics perspective, preventive prosthodontic care may therefore represent a cost‐effective approach by shifting expenditure from repeated replacement toward maintenance‐based continuity of care.

The relevance of preventive prosthodontics is particularly evident in geriatric oral healthcare. The NPHCE emphasizes preservation of functional independence, rehabilitation, and improvement in quality of life among older adults [35]. Because older individuals constitute a major population requiring removable, fixed, and implant‐supported prosthodontic rehabilitation, integration of prosthodontic maintenance, periodic oral functional assessment, nutritional counseling, and prevention of prosthesis‐related complications into geriatric healthcare pathways directly supports the objectives of healthy aging. Such integration may help address oral frailty, preserve masticatory efficiency, and improve nutritional and psychosocial outcomes in aging populations.

From an international perspective, the preventive philosophy of prosthodontics is closely associated with the World Health Organization Global Strategy on Oral Health (2023–2030), emphasizing life course approaches for oral health, incorporation of oral care services within primary health care systems, and lifelong maintenance of functional oral health outcomes [13, 14]. Preventive prosthodontics supports these efforts through a focus on the longevity of oral function, prevention of complications from prosthesis, and rehabilitation success through maintenance‐oriented models. The incorporation of prosthodontic maintenance indicators, recall systems, and preventive services for prosthesis within national oral health plans may assist in the achievement of these global objectives.

Although this review focuses on the Indian oral health policy framework, the preventive principles discussed are broadly applicable and may inform oral health policy development in other healthcare settings according to local needs and healthcare structures.

It should be noted that the integration of preventive prosthodontics in the healthcare system does not mean that new policy frameworks should be created for such purposes. Preventive prosthodontics can be integrated into the existing systems of oral health, primary healthcare, geriatrics, and universal health coverage. The integration of preventive prosthodontics can help maintain continuity of care and reduce treatment burden, increase the life of prosthesis, preserve oral function, and improve quality of life. Within this wider context, preventive prosthodontics is not simply a clinical concept for the discipline but rather an oral health approach for populations that can effectively connect restorative rehabilitation and prevention to public health.

## 6. Challenges and Barriers to Implementing Preventive Prosthodontics

Despite its strong conceptual foundation and growing clinical relevance, preventive prosthodontics has been inadequately applied in clinical practice and not adequately represented in the overall oral health care delivery system. Translation of preventive prosthodontic knowledge from theory into practice is hampered by various barriers existing at the level of the profession, education, systems, economics, patients, and policies.

The most important obstacle is the traditional view that prosthodontics is a discipline which is more concerned with replacing missing teeth rather than maintaining oral structures that have not been lost. The treatment approach in clinical practice often favors restoration after considerable structural loss, while not much effort is put into the interception of risk factors, maintenance of prosthetic health, and prevention of biological and mechanical complications [5, 12]. This paradigm of replacement leads to episodic intervention rather than prevention through continuity of care, hence increasing the burden of treatment over time.

Moreover, there are educational constraints that exacerbate this situation. Undergraduate and graduate‐level education in dentistry have focused on planning treatments, fabrication of prostheses, occlusion, and practical skills, while very little attention was paid to maintenance programs, recall schemes, risk‐oriented approach, and prevention of prosthesis‐associated complications. It means that clinicians can be well‐educated to perform prosthodontic rehabilitation but unprepared to apply preventive maintenance techniques to detect and manage early biological and mechanical complications [12, 18].

The limits in health systems constitute yet another significant barrier. In most health care organizations, prosthodontic care is still provided on a reactive basis, using treatment approaches without proper facilities for follow‐up in the long term. Large patient numbers, staffing shortages, poor recall systems, poor referral processes, and lack of standard protocols for maintenance protocols reduce opportunities for preventive monitoring of fixed, removable, and implant‐supported prostheses [13, 18, 20]. In resource‐constrained settings, priority is often directed toward immediate treatment needs, leaving preventive prosthodontic surveillance underdeveloped despite its potential long‐term benefits.

Reimbursement and economic issues could also stand in the way of the use of preventive prosthodontics. It is common that the financial system favors treatments and pays more attention to procedures rather than prevention, maintenance, follow‐ups, and early correction of minor dental prosthodontics problems. In publicly funded health‐care systems, outpatient maintenance‐oriented prosthodontic services often receive less policy attention than major rehabilitative procedures [16, 36]. This imbalance may inadvertently reinforce replacement‐based treatment models rather than incentivize continuity‐based preventive care, even when preventive approaches are likely to be more cost‐effective over time.

Further influences on implementation can be attributed to patient variables. Knowledge concerning the significance of proper maintenance of the prosthesis, regular recall appointments, good hygiene practices, and early reporting of any functional issues varies considerably. Patients tend to visit specialists once they experience considerable pain, prosthesis instability, tissue damage, or loss of function. Other factors that may impede the preventive care process include financial constraints, limited access, treatment fatigue, and lack of necessity of maintenance once the prosthesis is placed [1, 4].

In policy terms, preventive oral health care has increasingly become recognized in national and international health policies; yet prosthodontics have not been adequately discussed in any of these policies. In the current oral health policy frameworks, there is an emphasis on caries prevention, periodontal health, tobacco control, and disease detection, but there is little mention of systematic prosthodontics maintenance and prevention of complications resulting from prostheses and preservation of long‐term oral function [13, 14]. This omission limits institutional prioritization, funding allocation, and programmatic implementation of preventive prosthodontic services.

Despite the intimate relationship between these barriers, there is a low possibility of tackling all of them at once. Educational reform, raising awareness among professionals, and inclusion of the preventive concepts of prosthodontics in educational programs both at an undergraduate and post‐graduate level would be possible short‐term objectives that would require minimal funding while generating the necessary manpower for broader application. Clinical guidelines and maintenance strategies based on evidence could become mid‐term priorities with the objective of establishing clinical standards for prevention. On the other hand, inclusion in insurance and financing programs as well as national oral health policy formulation would necessitate long‐term policy work and collaboration with the government.

The successful adoption of preventive prosthodontics will need a collaborative effort by educators, clinicians, dental bodies, researchers, and policymakers. Promoting multidisciplinary collaboration, conducting good clinical research, establishing standardized maintenance procedures, and integrating preventive prosthodontics indicators into oral health policy and surveillance systems can aid in the adoption of preventive prosthodontics in clinical practice and public health programs. This collaborative approach will ensure that preventive prosthodontics is adopted from being just a concept in one branch of dentistry to becoming an essential part of sustainable oral health care.

## 7. Conclusion

Preventive prosthodontics is a significant change in current prosthodontic philosophy from a system that focuses more on replacing lost oral components to one that stresses preservation, early intervention, maintenance, and functional rehabilitation in the long run. Through the inclusion of preventive strategies throughout the course of care, prosthodontics will no longer remain confined to its traditional role of rehabilitation but will have a proactive role in preventing biological or mechanical problems, preservation of supporting oral tissues, and sustained maintenance of oral function.

In addition, the importance of preventive prosthodontics is not limited to just the clinical side. The principles of preventive prosthodontics coincide with the general aims of public health, such as promoting healthy aging, continuity of care, lifelong oral health, and sustainable healthcare provision. The incorporation of the methods of preventive prosthodontics into primary healthcare services, geriatric dentistry, recall programs, and healthcare financing will help achieve a better prosthesis survival rate, decrease the burden of care in the long run, increase patients’ quality of life, and support more equitable access to functional oral rehabilitation.

Although its relevance has grown over time, certain factors have contributed toward making difficult for it to be implemented in clinical settings. These include factors such as education, clinical guidance, reimbursement, and implementation of policies, among others. It will be necessary to bring together institutions, professional bodies, and the health system to implement these principles in models of care. Implementation of preventive prosthodontics principles will help make prosthodontic care more sustainable in the Indian oral health care system.

Ultimately, preventive prosthodontics serves as an important bridge between restorative rehabilitation and preventive oral healthcare. Recognition of this broader role may support the development of more sustainable, patient‐centered, and prevention‐oriented oral health systems capable of addressing the needs of aging populations and evolving global healthcare priorities.

## 8. Future Directions

Future research and policy development should focus on strengthening the evidence base and implementation pathways for preventive prosthodontics within clinical practice and public health systems. Several priority areas warranted particular attention.

There is a need for longitudinal clinical studies that can assess the impact of prevention‐focused protocols such as recall periods, maintenance procedures, and early complication management on the lifespan of the prosthetic devices, complications associated with prosthetics, patients’ health‐related outcomes, and the costs involved in their provision. Stronger outcome‐based evidence would support the integration of preventive maintenance strategies into routine prosthodontic care pathways.

The development of clinical guidelines to support preventive care using evidence is important. The standardization of guidelines that cover recall times, risk stratification, maintenance of fixed, removable, and implant‐supported prosthodontics, and the handling of prosthodontic complications can result in better implementation in different clinical environments.

An additional priority is the development of consensus‐based definitions for some basic terminologies in the field of prosthodontics like “preventive prosthodontic care” and “prosthodontic maintenance visit.” Now, these two terms are inconsistently utilized in the literature, creating barriers to conduct comparative studies and implementing any guidelines in the field. A consensus‐based definition of these terminologies will aid in developing more accurate research, improve communication among clinicians and policymakers, and support the integration of preventive prosthodontics into routine clinical practice and oral health policy. Such consensus‐building efforts could be led through collaboration among professional organizations such as the Indian Prosthodontic Society, the National Dental Commission, and international organizations, including WHO Collaborating Centers for Oral Health.

Reformation of the curriculum in the field of dentistry needs to integrate the principles of preventative prosthodontics as key competencies in undergraduate and postgraduate courses. An increased focus on maintenance planning, prosthesis surveillance, risk assessment, patient education, and continuity‐based approaches to care may prove more helpful for the future clinician in preventative prosthodontics.

Health system studies need to investigate the feasibility of possible methods of integration of preventive prosthodontics within the primary health care systems, as well as within geriatric oral health programs and universal health coverage schemes. It would be especially useful to conduct economic assessments on the cost effectiveness of maintenance‐oriented prosthodontics versus replacement‐oriented care.

Digital technologies are likely to play an increasingly important role in preventive prosthodontics through continuous monitoring, early detection of complications, and personalized maintenance strategies. Teledentistry could provide remote consultations that would allow the earlier detection of complications with prostheses, especially for elderly people or those who live in underserved communities [37]. Recall systems assisted by artificial intelligence would make it possible to personalize maintenance regimens depending on risk levels. Digital monitoring of prosthesis, electronic health records, and intraoral scanning technology could help with monitoring prosthesis performance and detecting biological and mechanical complications at an early stage [38]. Further studies could be carried out to investigate the viability and efficiency of using such technologies in preventive prosthodontics.

Future oral health policy should increasingly recognize maintenance of functional oral rehabilitation as an essential component of life‐course oral healthcare. The incorporation of preventive prosthodontic indicators into national oral health strategies, public health surveillance frameworks, and reimbursement systems may contribute to the development of more sustainable, prevention‐oriented oral healthcare models.

## Author Contributions

Conceptualization, methodology, investigation, data curation, formal analysis, writing – original draft: Bondada Venkata Mani Anirudh and Rodrigues Shobha Janette. Writing – review and editing: Bondada Venkata Mani Anirudh, Rodrigues Shobha Janette, and Mundathaje Mahesh.

## Funding

This research received no funding.

## Disclosure

All authors have read and approved the final version of the manuscript. The corresponding author had full access to all of the data in this study and takes complete responsibility for the integrity of the data and the accuracy of the data analysis. The restriction to English‐language publications may have introduced a language bias and could have resulted in the omission of relevant evidence published in other languages. In addition, study selection and thematic synthesis were performed by a single reviewer without independent duplicate screening. This may have introduced a selection bias and may limit the reproducibility of the review. This manuscript “Preventive Prosthodontics—The Overlooked Bridge Between Oral Health Policy and Disease Prevention” was presented as a poster at the 29th IAPHD National Conference 2025 Mangaluru held from November 28th to 30th, 2025 at Dr. TMA Pai International Convention Centre, Mangaluru, hosted by Manipal College of Dental Sciences Mangalore, Karnataka. All authors gave their consent to publish this manuscript.

## Ethics Statement

Ethical approval was not required for this study because it is a narrative review based exclusively on the published literature and publicly available policy documents. The study did not involve human participants, animals, or identifiable personal data.

## Consent

The authors have nothing to report.

## Conflicts of Interest

The authors declare no conflicts of interest.

## Supporting Information

Additional supporting information can be found online in the Supporting Information section.

## Supporting information


**Supporting Information** Table S1: The Scale for Assessment of Narrative Review Articles (SANRA) evaluation of the present narrative review, including the assessment of methodological quality and reporting characteristics.

## Data Availability

The data supporting the findings of this study are available from the corresponding author upon reasonable request.
