# Supplementary material for: Preventive Prosthodontics: Integrating Preventive Prosthodontic Principles Into the Indian Oral Health Policy Framework
Source: Int J Dent. 2026 Oct 20;2026:7265763. doi: 10.1155/ijod/7265763 (PMC13682850; doi:10.1155/ijod/7265763)
Supplement: Supplementary file 1 [file IJOD-2026-7265763-s001.docx]

**Supplementary Table S1. SANRA (Scale for the Assessment of Narrative Review Articles) Assessment of the Present Narrative Review**

| SANRA Item | Score (0–2) | Justification |
| --- | --- | --- |
| Justification of the article's importance for the readership | 2 | The introduction establishes the clinical and policy relevance of preventive prosthodontics and identifies an important gap in oral health policy. |
| Statement of concrete aims or formulation of questions | 2 | The objectives clearly state the scope of the review, including the concept, levels of prevention, and policy integration of preventive prosthodontics. |
| Description of the literature search | 2 | The literature search strategy, databases, search period, keywords, eligibility criteria, and study selection process are described in the Methods section. |
| Referencing | 2 | Key statements are supported by appropriate references from peer-reviewed literature and policy documents. |
| Scientific reasoning | 2 | The manuscript synthesizes evidence from prosthodontics, preventive dentistry, and public health to develop a coherent conceptual framework. |
| Appropriate presentation of data | 2 | Findings are presented in a structured manner, including thematic sections and a summary table linking preventive interventions with health system implications. |
| Total Score | **12/12** |  |
